# Headspace Solid-Phase Microextraction and Ultrasonic Extraction with the Solvent Sequences in Chemical Profiling of *Allium ursinum* L. Honey

**DOI:** 10.3390/molecules22111909

**Published:** 2017-11-06

**Authors:** Igor Jerković, Piotr M. Kuś

**Affiliations:** 1 Department of Organic Chemistry, Faculty of Chemistry and Technology, University of Split, Rudera Boskovica 35, 21000 Split, Croatia; 2 Department of Pharmacognosy, Wroclaw Medical University, ul. Borowska 211a, 50-556 Wroclaw, Poland; kus.piotrek@gmail.com

**Keywords:** *Allium ursinum* L. honey, headspace solid-phase microextraction (HS-SPME), ultrasonic solvent extraction (USE) with the solvent sequence, (*E*)-4-(r-1′,t-2′,c-4′-trihydroxy-3′,6′,6′-trimethylcyclohexyl)-but-3-en-2-one, hydroquinone, methyl syringate, 4-hydroxybenzoic acid

## Abstract

A volatile profile of ramson (wild garlic, *Allium ursinum* L.) honey was investigated by headspace solid-phase microextraction (HS-SPME) and ultrasonic solvent extraction (USE) followed by gas chromatography and mass spectrometry (GC-FID/GC-MS) analyses. The headspace was dominated by linalool derivatives: *cis*- and *trans*-linalool oxides (25.3%; 9.2%), hotrienol (12.7%), and linalool (5.8%). Besides direct extraction with dichloromethane and pentane/diethyl ether mixture (1:2, *v*/*v*), two solvent sequences (I: pentane → diethyl ether; II: pentane → pentane/diethyl ether (1:2, *v*/*v*) → dichloromethane) were applied. Striking differences were noted among the obtained chemical profiles. The extracts with diethyl ether contained hydroquinone (25.8–36.8%) and 4-hydroxybenzoic acid (11.6–16.6%) as the major compounds, while (*E*)-4-(r-1′,t-2′,c-4′-trihydroxy-2′,6′,6′-trimethylcyclohexyl)but-3-en-2-one predominated in dichloromethane extracts (18.3–49.1%). Therefore, combination of different solvents was crucial for the comprehensive investigation of volatile organic compounds in this honey type. This particular magastigmane was previously reported only in thymus honey and hydroquinone in vipers bugloss honey, while a combination of the mentioned predominant compounds is unique for *A. ursinum* honey.

## 1. Introduction

Ramson (wild garlic, *Allium ursinum* L.) is a perennial plant, widely distributed in Europe. Phytochemical investigations of this plant revealed the presence of *S*-alk(en)yl-l-cysteine-sulfoxides (methiin, alliin, isoalliin, propiin, and ethiin) and their degradation products ((poly)sulfides, dithiins, or ajoenes) [1,2]. Apart from the abovementioned, various sulphur compounds have also been detected as constituents of its essential oil, e.g., disulfides, trisulfides, and tetrasulfides [3,4]. *A. ursinum* has been also reported to be a rich source of phenolic compounds (up to 27.9 g GAE (gallic acid equivalent)/100 g) [5]. Similar to organosulfur compounds, it was found to contain steroidal saponins that are also commonly found in the *Allium* genus [1,6]. Other identified constituents of interest include lectins, polysaccharides, and fatty acids [1]. A great number of in vitro and in vivo experiments showed that *A. ursinum* is a plant with antimicrobial, cytotoxic, antioxidant, and cardio-protective effects [1,7].

*A. ursinum* provides excellent spring bee pasture with a good nectar flow [8,9]. *Allium* species tend to secrete highly concentrated nectar, and the daily nectar production of *A. ursinum* ranged from 0.1 to 3.8 µL per flower, with sugar concentrations of 25% to 50%. However, the floral nectar volume and concentration varies in different populations of *A. ursinum* which can be also strongly affected by the varying conditions in different natural habitats. Nevertheless, the honey cannot be produced on a regular basis and its production is limited [8].

In continuation of the chemical fingerprinting of different unifloral honey types in search of specific or nonspecific chemical markers of botanical origin, the focus of this work was on not yet investigated volatile organic compounds (VOCs) of *Allium ursinum* L. honey of Croatian origin (a very rare sample). Headspace solid-phase microextraction (HS-SPME) followed by gas chromatography and mass spectrometry (GC-FID/GC-MS) analysis was applied to investigate its headspace chemical profile. To complement the honey profiling with data on less volatile organic compounds, ultrasonic solvent extraction (USE) was applied with solvents of different polarities, and the obtained extracts were analysed by GC-FID/GC-MS.

## 2. Results

A rare sample of *A. ursinum* honey from Croatia was confirmed to be unifloral according to performed mellisopalynological analysis. It contained 58% of *Allium ursinum* L. pollen grains accompanied by the pollen from *Prunus* spp. (19%), *Acer* spp. (14%), and a minor contribution from the grains of *Salix* spp. (2%), *Fraxinus excelsior* (1%), *Tilia* spp. (1%), Asteraceae (1%), Ericaceae (1%), and Brassicaceae (1%).

At the time of blooming, *A. ursinum* plants emit a strong garlic odour that can also be smelled in the nectar and in front of the beehives. However, it has been reported that the odour of the corresponding ripe honey is different, with a pleasant, particular aroma [8]. Therefore, significant differences among the chemical profiles obtained from *A. ursinum* honey VOCs and the corresponding plant VOCs were expected. To investigate in detail the headspace, volatile, and semi-volatile compounds from *A. ursinum* honey, up to-date complementary methodologies were applied: headspace solid-phase microextraction (HS-SPME) and ultrasonic solvent extraction (USE) followed by GC-FID/GC-MS analyses. Striking differences were found among the chemical profiles obtained by those methods and the plant VOCs.

### 2.1. The Headspace Chemical Profile

The headspace of *A. ursinum* honey (Table 1) dominated with monoterpenes—linalool derivatives such as *cis*- and *trans*-linalool oxides (9.2%; 25.3%), hotrienol (12.7%), and linalool (5.8%).

Few benzene derivatives, often found in different honey types [10], were detected by HS-SPME with minor abundance, e.g., benzaldehyde (1.1%), phenylacetaldehyde (1.7%), 2-phenylethanol (3.0%), 4-methoxybenzaldehyde (1.1%), and phenylacetonitrile (1.9%). 4-Ketoisophorone (2.8%) was the only norisoprenoid detected in the headspace in distinction from the extracts. Dimethyl disulfide (1.2%) was the only headspace compound that could be connected to the plant VOCs (it was found in *A*. *ursinum* essential oil). The majority of the essential oil constituents, such as typical sulphides, disulfides, and trisulfides, were not present in the honey [3,4]. As was mentioned before, ripe ramson honey possesses a pleasant, particular aroma, and the probably typical sulfur volatile organic compounds were lost during the honey maturation in the hive. In addition, it is well known that honey VOCs usually significantly differ from the corresponding plant VOCs [11].

### 2.2. The Extracts Chemical Profile

Ultrasonic extraction (USE) of the honey was first performed separately with two solvents: (a) the mixture of pentane and diethyl ether (1:2, *v*/*v*) (A), and (b) dichloromethane (B), as in our previous research [12,13]. Significant differences were found among chemical profiles of the extracts (Table 2). The extract A contained 1,4-benzenediol (25.8%) as the major compound followed by a variety of benzene derivatives, particularly benzoic acid and its *p*-substituted derivatives: 4-hydroxybenzoic acid (16.4%), benzoic acid (4.4%), and 4-methoxybenzoic acid (3.7%). 4-Hydroxybenzaldehyde (10.3%) and methyl syringate (9.8%) were also quite abundant. In contrast, the extract B contained as the major compound C_13_-norisoprenoid (*E*)-4-(r-1′,t-2′,c-4′-trihydroxy-3′,6′,6′-trimethylcyclohexyl)-but-3-en-2-one (18.3%), which was present only with 3.1% in the extract A. Aromatic compounds were present among the major compounds (similar as in the extract A: methyl syringate (12.2%), 4-hydroxybenzaldehyde (9.9%), 4-methoxybenzoic acid (4.2%), and benzoic acid (3.1%)). However, the major difference in 4-hydroxybenzoic acid and hydroquinone abundance was noted among two extracts (dominated in A). Both of them also contained other C_13_-norisoprenoids (solvent A; solvent B): 3-oxo-α-ionone (1.8%; 1.5%), vomifoliol (1.1%; 2.6%), and 3-oxo-7,8-dihydro-α-ionone (0.5%; 0.1%). Higher aliphatic compounds were present among minor constituents in both extracts as well as *trans*- or *cis*-linalool oxides (furan type).

Since significant differences were found in the obtained chemical profiles, previously applied USE was modified and two solvent sequences (sequence I: pentane (C) → diethyl ether (D); sequence II: pentane (C) → pentane:diethyl ether (1:2, *v*/*v*) (E) → dichloromethane (F)) were applied for the honey extraction and more complete profiling by the fractionation of compounds according to their distribution among the solvents of different polarities. Pentane extract (C) contained methyl syringate (26.2%) and docosane (23.0%) as the major compounds. *cis*- and *trans*-Linalool oxides (furan type) were the most abundant among all extracts (3.3%; 1.2%). 3-Hydroxy-4-phenylbutan-2-one was only present in this extract (2.8%). Higher aliphatic compounds were also present (Table 2). However typical compounds found in direct extracts with solvents A and B were not present. Diethyl ether extract (sequence I, D) contained hydroquinone (36.8%) and 4-hydroxybenzoic acid (16.6%) as the major constituents. (*E*)-4-(r-1′,t-2′,c-4′-trihydroxy-3′,6′,6′-trimethylcyclohexyl)-but-3-en-2-one was present with 6.2%, methyl syringate with 3.0%, and 4-hydroxybenzaldehyde with 5.3%. Other C_13_-norisoprenoids were found with minor abundance, such as 3-oxo-α-ionone, 3-oxo-7,8-dihydro-α-ionone, and vomifoliol. Only *trans*-linalool oxide (furan type) was found. It can be seen that this extract was purified from less polar compounds by previous extraction with pentane (sequence I). The extract with pentane:diethyl ether (1:2, *v*/*v*) applied in sequence II (E) contained as major compounds 1,4-benzenediol (27.7%), docosane (14.0%), 5-hydroxymethylfurfural (13.3%), 4-hydroxybenzoic acid (11.6%), methyl syringate (6.6%), and (*E*)-4-(r-1′,t-2′,c-4′-trihydroxy-3′,6′,6′-trimethylcyclohexyl)-but-3-en-2-one (3.3%). Other C_13_-norisoprenoids (3-oxo-α-ionone, 3-oxo-7,8-dihydro-α-ionone, and vomifoliol) were present. Lot of similarities were noted among diethyl ether extract in sequence I (D) and the extract with the mixture of pentane:diethyl ether (1:2, *v*/*v*) in sequence II (E) regarding the distribution of hydroquinone, 4-hydroxybenzoic acid, 4-hydroxybenzaldehyde, and C_13_-norisoprenoids. The major difference was the abundance of docosane in the extract E (sequence II). Since dichloromethane extract in sequence II (F) was applied after pentane extraction and after the extraction with pentane:diethyl ether (1:2, *v*/*v*), it was expected to contain the least compounds of all the extracts (Table 2). However, this extract was dominated by (*E*)-4-(r-1′,t-2′,c-4′-trihydroxy-3′,6′,6′-trimethylcyclohexyl)-but-3-en-2-one (49.1%), which could be useful for its isolation from the honey matrix. Such a result was also expected and it is in accordance with the data obtained from the direct extraction with dichloromethane. It is interesting to note that other C_13_-norisoprenoids were extracted with pentane:diethyl ether (1:2, *v*/*v*) previously applied in sequence II (E), and they were not present in dichloromethane extract (F). Docosane was the second major compound (25.9%) in this extract, followed by (*Z*)-octadec-9-en-1-ol (8.9%) and octadecan-1-ol (2.8%).

In comparison with HS-SPME (Table 1 and Table 2), only a few compounds were similar, while linalool and its derivatives were found with significantly lower abundance in the extracts than in the headspace. Epoxidation of linalool gives 6,7-epoxylinalool, which undergoes further reactions to form linalool oxides, while hotrienol is derived from hydroxylated linalool derivatives [11]. Higher abundance of linalool, *cis*-, and *trans*-linalool oxide were found in the headspace of *Coriandrum sativum* L. [14] and *Citrus* spp. [13,15,16] honey types. Regarding the extract chemical profiles, no major similarity was found among the profiles of other honey types. A combination of predominant compounds (*E*)-4-(r-1′,t-2′,c-4′-trihydroxy-3′,6′,6′-trimethylcyclohexyl)-but-3-en-2-one, hydroquinone, methyl syringate, and 4-hydroxybenzoic acid is unique to *A. ursinum* honey. 1,4-Dihydroxybenzene was proposed as a floral marker compound for vipers bugloss (*Echium vulgare* L.) honey [17]. High proportions of benzoic acid and its derivatives were found in *Salix* spp. honeydew extractives [18], but with a minor percentage of 4-hydroxybenzoic acid. The latter was found abundant by HPLC in buckwheat (*Fagopyrum esculentum* L.) honey [19]. (*E*)-4-(r-1′,t-2′,c-4′-trihydroxy-2′,6′,6′-trimethylcyclohexyl)but-3-en-2-one contains a megastigmane structure. Structurally, megastigmanes are C_13_-carbon skeleton compounds, which are commonly classified as C_13_-norisoprenoids, also assumed to be apocarotenoides. Megastigmanes possess a unique basic skeleton with a six-membered ring with a double bond within the ring system, followed by methyl and dimethyl substitutions and an attached four membered chain with a double bond in the *trans*-mode [20]. The biosynthesis of this compound can be envisaged as proceeding via the alkene with a double bond within the ring system and via one or both of the epoxides [20]. Although a wide variety of degraded carotenoid-like substances have been identified from different honey types [13], this appears to be a rare situation where a trihydroxy ketone has been found. In fact, it was previously isolated and characterized by X-ray crystallographic analysis as a dominant substance from the ether extracts of New Zealand thyme honey [21]. Its recorded MS spectra were *m*/*z* 224 (6%), 141 (9), 140 (8), 125 (55), 124 (l2), 123 (18), 109 (8), 99 (7), 97 (23), 95 (6), 83 (9), 71 (13), 69 (8), 55 (17), 43 (96) and the reported data [21] on MS of (*E*)-4-(r-1′,t-2′,c-4′-trihydroxy-3′,6′,6′-trimethylcyclohexyl)-but-3-en-2-one were *m*/*z* 224 (6%), 141 (9), 140 (8), 125 (43), 124 (l0), 123 (17), 109 (8), 99 (7), 97 (23), 95 (6), 83 (9), 71 (13), 69 (8), 55 (17), 43 (100). This compound exerted significant apoptotic activity in PC-3 prostate cancer cells at 100 μM, while it inhibited NF-κB phosphorylation and IL-6 secretion at a concentration range of 10^−6^–10^−4^ M [22].

## 3. Materials and Methods

A rare and representative *Allium ursinum* L. honey sample was collected from a professional beekeeper in Croatia (more unifloral samples were not available). The sample was stored in a hermetically closed glass bottle at 4 °C until the volatiles were isolated. Melissopalynological analysis was performed according to the International Commission for Bee Botany [23]. Microscopical examination was carried out on a Hund H 500 light microscope (Helmut Hund GmbH, Wetzlar, Germany) attached to a digital camera (Motic m 1000, Motic Deutschland GmbH, Wetzlar, Germany) and coupled to an image analysis system (Motic Images Plus software, Motic Deutschland GmbH) for the morphometry of pollen grains.

### 3.1. Headspace Solid-Phase Microextraction (HS-SPME)

The headspace solid-phase extraction (HS-SPME) was performed using a manual SPME holder using polydimethylsiloxane/divinylbenzene (PDMS/DVB) fiber that was conditioned prior to the usage according to Supelco (Bellefonte, PA, USA) instructions. The honey/saturated water solution (5 mL, 1:1 (*v*/*v*); saturated with NaCl) was placed in a 15-mL glass vial and hermetically sealed with polytetrafluorethylene (PTFE)/silicone septa. The vial was maintained in a water bath at 60 °C during equilibration (15 min) and HS-SPME (45 min) under constant stirring (1000 rpm) with a magnetic stirrer, and the sample was kept below the water level of the water bath. After sampling, the SPME fiber was withdrawn into the needle, removed from the vial, and inserted into the injector (250 °C) of the GC-FID and GC-MS for 6 min, where the extracted volatiles were thermally desorbed directly to the GC column. The experiment was performed in triplicate.

### 3.2. Ultrasonic Solvent Extraction (USE)

Ultrasound-assisted solvent microextraction (USE) was performed in an ultrasound cleaning bath (Clean 01, MRC Scientific Instruments, London, UK) by the indirect sonication mode at a frequency of 37 kHz at 25 ± 3 °C. The advantage of using USE is the isolation of volatile and semi-volatile as well as water-soluble organic compounds without the application of heat. Different solvents were used for USE: a mixture of pentane/diethyl ether, 1:2 (*v*/*v*), dichloromethane, pentane, and diethyl ether. The mixture and dichloromethane were separately used for the extractions. A previously developed USE method was modified with the solvent sequences that were applied for the honey extraction. Sequence I consisted of the extraction with pentane followed by the extraction with diethyl ether (pentane → diethyl ether). Sequence II consisted of pentane extraction followed by the extraction with pentane:diethyl ether 1:2 (*v*/*v*) and afterwards with dichloromethane (pentane → pentane:diethyl ether 1:2 (*v*/*v*) → dichloromethane). For each extraction, 40 grams of the honey was dissolved in distilled water (22 mL) in a 100-mL flask. Magnesium sulfate (1.5 g) was added and vortexed (10 min). The solvent volume was 20 mL and the sonication was applied for 30 min. After the sonication, the organic layer was separated by centrifugation and filtered over anhydrous MgSO_4_. The aqueous layer was returned to the flask and another batch of the same extraction solvent was added and extracted for 30 min. The organic layer was separated in the same way as the previous layer and filtered over anhydrous MgSO_4_, and the aqueous layer was sonicated a third time for 30 min with another batch of the extraction solvent. Combined organic extracts were concentrated to 0.2 mL by distillation with a Vigreaux column, and 1 µL was used for GC-FID/GC-MS analyses. The experiments were performed in triplicate.

### 3.3. GC-FID and GC-MS Analyses

The GC-FID analyses were conducted with an Agilent Technologies (Palo Alto, CA, USA) gas chromatograph model 7890A equipped with a flame ionization detector (FID) and a HP-5MS capillary column (5% phenyl-methylpolysiloxane, Agilent J and W, Santa Clara, CA, USA). The GC conditions were described previously [13,18]. In brief, the oven temperature was programmed isothermal at 70 °C for 2 min, increasing from 70–200 °C at 3 °C·min^−1^, and held isothermally at 200 °C for 15 min; the carrier gas was He (1.0 mL·min^−1^); and the total run time was 65 min.

The GC-MS analyses were conducted with an Agilent Technologies (Palo Alto, CA, USA) gas chromatograph model 7820A equipped with a mass selective detector (MSD) model 5977E (Agilent Technologies) and a HP-5MS capillary column, under the same conditions as those described for the GC-FID analysis. The MSD (EI mode) was operated at 70 eV, and the mass range was 30–300 amu, as previously reported [13].

The identification was based on the comparison of VOC retention indices (RI), determined relative to the retention times of a homologous series of *n*-alkanes (C_9_–C_25_), with those reported in the literature and their mass spectra with authentic compounds available in our laboratories or those listed in Wiley 9 (Wiley, New York, NY, USA) and NIST 14 (D-Gaithersburg) mass spectral libraries. The percentage composition of the samples was computed from the GC peak areas using the normalization method (without correction factors). The average component percentages in Table 1 and Table 2 were calculated from duplicate GC-FID and GC-MS analyses.

## 4. Conclusions

The unusual chemical profile of *A. ursinum* honey was investigated and described for the first time. The headspace was dominated by linalool and its derivatives, which is not specific. The extracts showed remarkable variabilities according to the solvents applied, which is important to point out since the use of only one solvent could lead to incomplete results for *A. ursinum* honey. Namely, the extracts obtained with diethyl ether as the solvent contained 1,4-benzenediol and 4-hydroxybenzoic acid as the major compounds, while (*E*)-4-(r-1′,t-2′,c-4′-trihydroxy-2′,6′,6′-trimethylcyclohexyl)but-3-en-2-one predominated in the dichloromethane extracts. The applied sequence of solvents enabled the fractionation of the compounds according to polarity, and sequence II was useful for the concentration and possible isolation of (*E*)-4-(r-1′,t-2′,c-4′-trihydroxy-2′,6′,6′-trimethylcyclohexyl)but-3-en-2-one. More samples should be investigated to confirm these compounds as characteristic of this honey type.

## Figures and Tables

**Table 1:** The Headspace volatiles of the sample determined by HS-SPME/GC-MS.

No.	Compound	RI ^1^	RI ^2^	% ^3^	No	Compound	RI ^1^	RI ^2^	% ^3^
1	Dimethyl disulfide	<900	747	1.2	14	Hotrienol	1106	1110	12.7
2	Butanoic acid	<900	763	0.7	15	2-Phenylethanol ^4^	1116	1116	3.0
3	3-Methylbut-2-enal *	<900	781	1.0	16	Phenylacetonitrile	1143	1141	1.9
4	Octane	<900	800	0.1	17	4-Ketoisophorone	1147	1147	2.8
5	3-Methylbutanoic acid	<900	888	1.7	18	Octanoic acid ^4^	1176	1179	1.7
6	Benzaldehyde ^4^	965	966	1.1	19	Nonan-1-ol ^4^	1178	1171	1.4
7	Hexanoic acid ^4^	980	982	0.9	20	*trans*-Linalool oxide (pyran type)	1183	1183	1.5
8	(*E*)-Hex-3-enoic ^4^ acid	991	/	0.7	21	α-Terpineol ^4^	1194	1191	0.8
9	(*Z*)-Hex-3-enoic acid	1013	1013	0.8	22	5-Hydroxymethylfurfural ^4^	1230	1226	4.0
10	Phenylacetaldehyde ^4^	1048	1049	1.7	23	4-Methoxybenzaldehyde ^4^	1256	1258	1.1
11	*cis*-Linalool oxide (furan type)	1076	1075	25.3	24	Nonanoic acid ^4^	1273	1276	4.0
12	*trans*-Linalool oxide (furan type)	1091	1091	9.2	25	3,4,5-Trimethylphenol **	1336	-	3.2
13	Linalool ^4^	1101	1101	5.8	26	Hexadecanoic acid ^4^	1970	1977	1.7

^1^ RI—retention indices on HP-5MS column relative to C_9_–C_25_ alkanes; ^2^ RI from the literature (National Institute of Standards and Technology (NIST) Chemistry WebBook, NIST Standard Reference Database Number 69, http://webbook.nist.gov/chemistry/); ^3^ Area percentages (%), ^4^ identification confirmed with standard compound; *—tentatively identified; **—correct isomer is not identified.

**Table 2:** The volatile organic compounds composition of the sample determined by ultrasonic solvent extraction (USE)/GC-FID; GC-MS.

No.	Compound	RI ^1^	RI ^2^	A	B	C	D	E	F
				% ^3^	% ^3^	% ^2^	% ^3^	% ^3^	% ^3^
1	2-Furancarboxaldehyde	<900	835	-	-	-	-	0.6	0.1
2	4-Methyloctane	<900	/	0.1	-	0.1	0.1	-	-
3	1,3-Dimethylbenzene **	<900	864	1.5	-	0.6	0.7	0.6	-
4	2-Furanmethanol	<900	866	-	-	-	-	0.1	-
5	Ethylbenzene	<900	868	0.2	-	0.6	0.2	0.1	-
6	3-Methylbutanoic acid (Isovaleric acid)	<900	888	-	-	-	-	0.1	-
7	3-Methylbut-2-enoic acid *	<900	/	0.1	0.2	-	0.1	0.1	-
8	Ethenylbenzene	<900	892	0.1	-	-	0.1	-	-
9	1,2-Dimethylbenzene **	<900	897	0.3	-	0.8	0.1	-	-
10	Methoxybenzene	912	/	0.1	-	-	0.2	-	-
11	2-Acetylfuran	918	914	-	-	-	-	0.1	-
12	Benzaldehyde ^4^	965	966	0.1	0.2	0.7	0.1	-	-
13	5-Methylfurfural ^4^	970	966	-	-	-	-	0.1	-
14	(*E*)-Hex-3-enoic acid ^4^	991	/	0.5	0.3	-	0.2	-	-
15	(Z)-Hex-3-enoic acid	1013	1013	0.1	0.2	-	0.1	0.1	-
16	Pantolactone	1044	/	0.1	0.2	-	0.1	0.1	0.2
17	Phenylacetaldehyde ^4^	1048	1049	0.1	0.2	0.6	0.1	-	-
18	Acetophenone ^4^	1065	1065	-	-	-	0.1	-	-
19	*cis*-Linalool oxide (furan type)	1076	1075	0.7	0.3	3.3	0.2	1.3	0.1
20	*trans*-Linalool oxide (furan type)	1091	1091	0.2	0.2	1.2	-	0.4	-
21	Linalool ^4^	1102	1101	0.1	-	-	-	-	-
22	Hotrienol	1106	1110	0.1	0.2	0.6	-	-	-
23	2-Phenylethanol ^4^	1116	1116	0.7	0.7	1.6	0.3	0.4	-
24	2,3-Dihydro-3,5-dihydroxy-6-methyl-4*H*-pyran-4-one	1143	1149	0.2	0.3	-	0.3	-	-
25	Benzoic acid ^4^	1181	1178	4.4	3.1	2.0	2.2	1.2	0.3
26	Terpendiol I	1191	1191	0.7	0.5	0.7	0.3	-	-
27	5-Hydrohymethylfurfural ^1^	1230	1226	0.8	2.6	-	1.1	13.3	0.8
28	4-Methoxybenzaldehyde ^4^	1259	1258	0.1	0.2	0.7	-	0.3	-
29	Phenylacetic acid ^4^	1269	1270	0.8	0.8	-	0.6	0.1	-
30	Nonanoic acid ^4^	1273	1276	0.1	0.2	0.7	-	-	-
31	1,4-Benzenediol ^4^ (Hydroquinone)	1328	/	25.8	2.4	-	36.8	27.7	0.7
32	3,4,5-Trimethylphenol **	1336	1331	0.3	0.5	1.6	0.3	-	0.2
33	3-Hydroxy-4-phenylbutan-2-one	1354	1348	-	-	2.8	-	-	-
34	Phenylpropanoic acid ^4^	1359	1361	1.8	1.6	-	0.4	-	-
35	1-Hydroxylinalool **	1365	/	0.3	0.3	-	-	0.1	-
36	4-Hydroxybenzaldehyde ^4^	1393	/	10.3	9.9	-	5.3	2.5	1.2
37	4-Hydroxy-3-methoxy-benzaldehyde (Vanillin) ^4^	1412	1394	0.3	0.7	-	-	-	-
38	4-Methoxybenzoic acid (*p*-Anisic acid) ^4^	1452	1451	3.7	4.2	-	2.5	0.7	0.4
39	(*E*)-3-Phenylprop-2-enoic acid (*trans*-Cinnamic acid) ^4^	1455	1457	0.9	0.7	-	0.4	0.1	0.1
40	Methyl 4-hydroxybenzoate ^4^	1482	/	0.2	0.3	-	-	-	-
41	4-Hydroxy-phenylacetonitrile *	1502	/	1.0	1.3	-	0.8	0.3	0.3
42	Methyl 4-hydroxy-3-methoxybenzoate	1530	1527	0.2	0.2	-	-	-	-
43	4-Hydroxybenzoic acid ^4^	1558	1558	16.4	0.2	-	16.6	11.6	-
44	3,5,5-Trimethyl-4-(3-oxo-1-butenyl)cyclohex-2-en-1-one (3-Oxo-α-ionone)	1665	1661	1.8	1.5	2.4	-	0.3	-
45	Syringaldehyde ^4^	1668	1667	-	0.7	-	-	0.1	-
46	3,5,5-Trimethyl-4-(3-oxobutyl)cyclohex-2-en-1-one (3-Oxo-7,8-dihydro-α-ionone)	1682	1681	0.5	0.1	0.7	0.3	0.3	-
47	Heptadecane ^4^	1700	1700	0.2	-	0.9	0.1	-	-
48	Methyl syringate ^4^	1744	1744	9.8	12.2	26.2	3.0	6.6	1.0
49	4-Hydroxy-3,5,5-trimethyl-4-(3-oxo-1-butenyl)cyclohex-2-en-1-one (Vomifoliol)	1802	1796	1.1	2.6	-	1.1	0.9	-
50	Hexadecan-1-ol ^4^	1882	1883	0.1	0.2	1.2	0.2	0.4	1.5
51	(*E*)-4-(r-1′,t-2′,c-4′-trihydroxy-3′,6′,6′-trimethylcyclohexyl)-but-3-en-2-one	1960	/	3.1	18.3	-	6.2	3.3	49.1
52	Hexadecanoic acid ^4^	1970	1977	1.1	4.1	1.8	0.7	0.1	0.1
53	(*Z*)-Octadec-9-en-1-ol ^4^	2060	2060	0.8	6.2	3.1	2.0	0.1	8.9
54	Octadecan-1-ol ^4^	2084	2081	0.1	1.5	0.8	0.2	2.2	2.8
55	(*Z*)-Octadec-9-enoic acid ^4^	2142	2140	1.5	2.4	2.8	1.7	0.1	0.1
56	Docosane ^4^	2200	2200	0.1	1.0	23.0	0.2	14.0	25.9
57	Tricosane ^4^	2300	2300	0.7	1.0	4.3	0.7	0.1	0.1

^1^ RI—retention indices on HP-5MS column relative to C_9_–C_25_ alkanes; ^2^ RI from the literature (NIST Chemistry WebBook, NIST Standard Reference Database Number 69, http://webbook.nist.gov/chemistry/); ^3^ Area percentages (%); ^4^ identification confirmed with standard compound; *—tentatively identified; **—correct isomer is not identified; - indicates that compound is not identified. **A**—USE with pentane:diethyl ether (1:2, *v*/*v*); **B**—USE with dichloromethane; **C**—sequence I/II: USE with pentane; **D**—sequence I: USE with diethyl ether after C (pentane extraction), **E**—sequence II: USE with the mixture pentane:diethyl ether (1:2, *v*/*v*) after C (pentane extraction); **F**—sequence II: USE with dichloromethane after E (the extraction with the mixture pentane:diethyl ether (1:2, *v*/*v*)) and C (pentane extraction).
